# Supplementary material for: Cell Painting-based bioactivity prediction boosts high-throughput screening hit-rates and compound diversity
Source: Nat Commun. 2024 Apr 24;15:3470. doi: 10.1038/s41467-024-47171-1 (PMC11043326; doi:10.1038/s41467-024-47171-1)
Supplement: Supplementary file 1 — Supplementary information [file 41467_2024_47171_MOESM1_ESM.docx]

**Supplementary figures:**


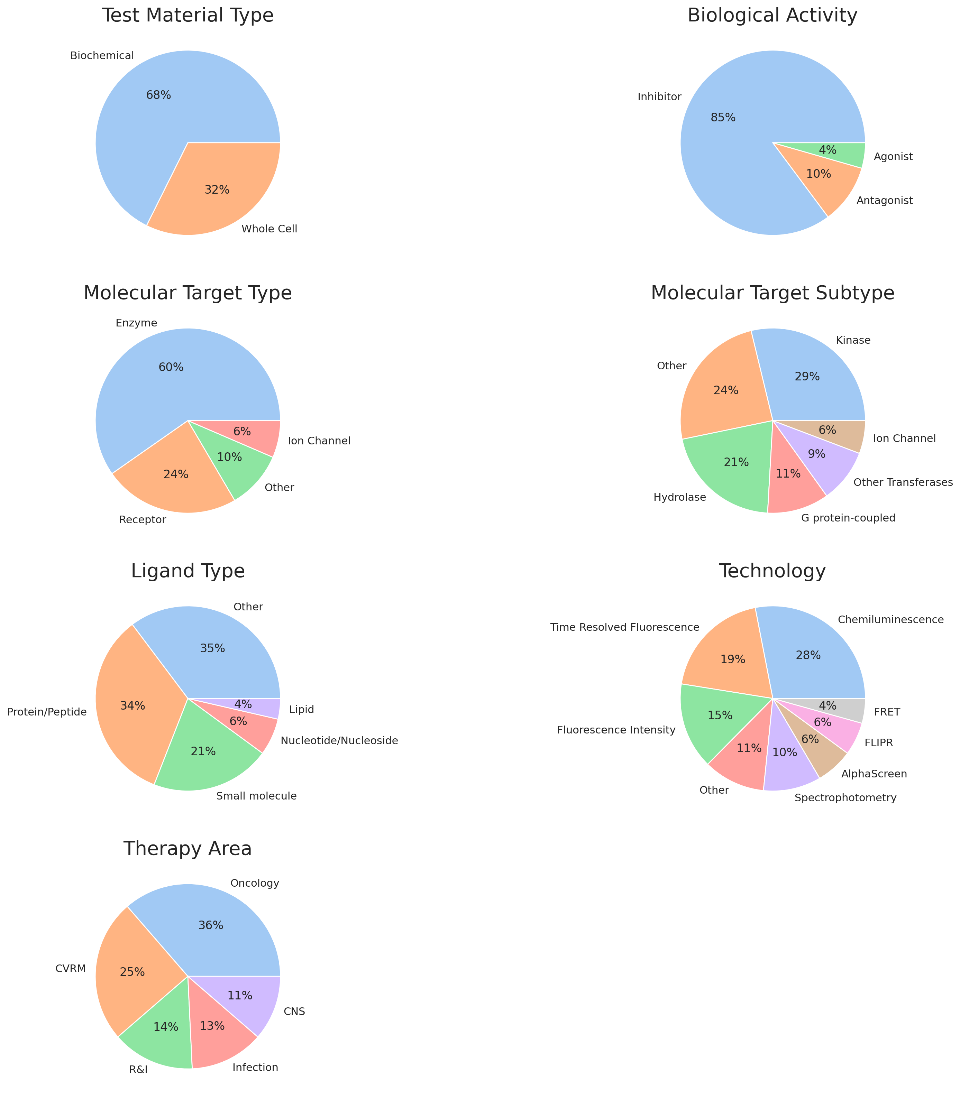


**Supplementary Figure 1 – HTS assay characteristics.** Pie-chars of assay characteristics of the HTS assay used. Grouped by major classes, rare characteristics were added into the other group.


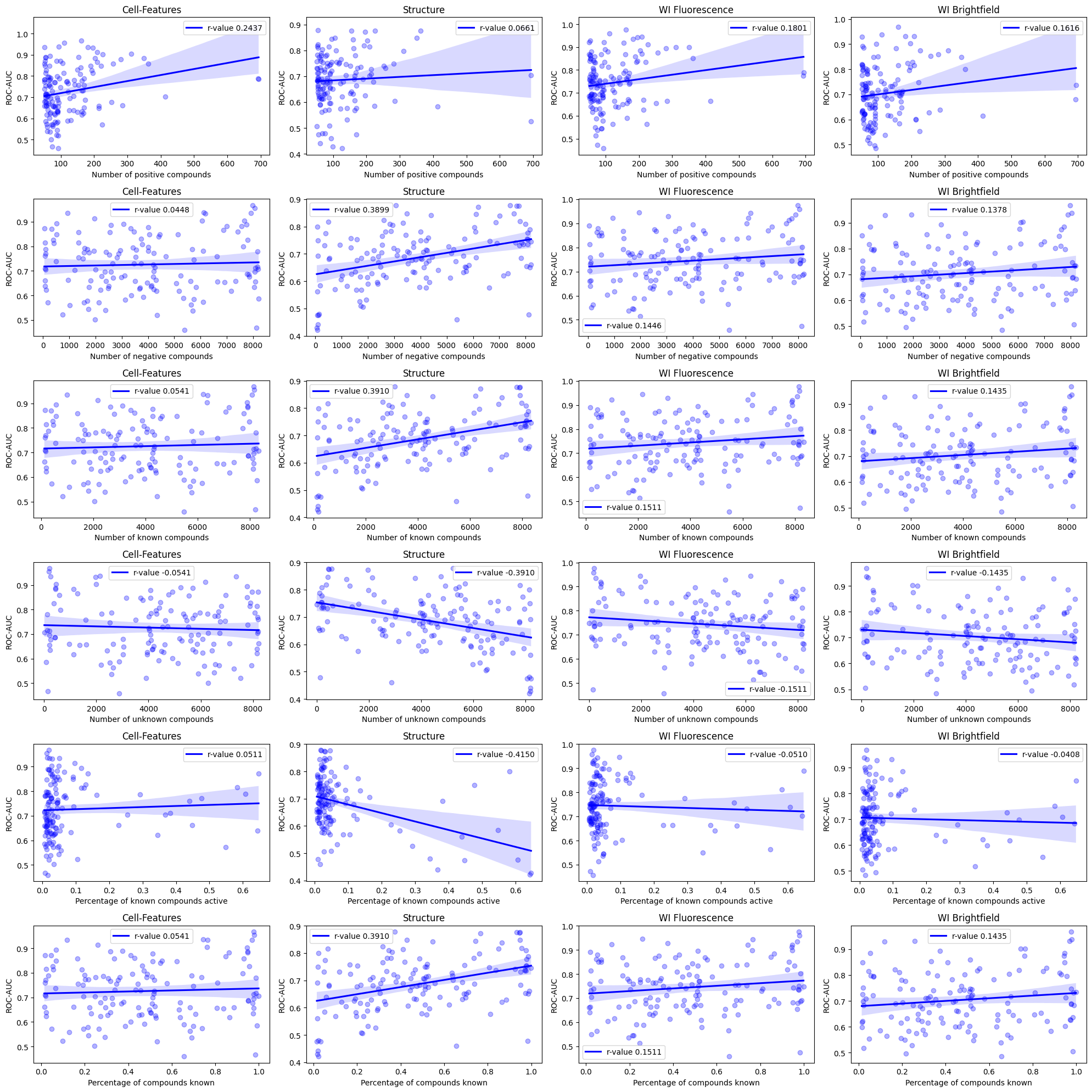


**Supplementary Figure 2 – Model performance vs. HTS data availability.** Modality-wise performance per assay vs. HTS assay data point availability (n=140 assays). Each column represents one input modality, rows represent, 1.) The number of known active compounds, 2.) number of known inactive compounds, 3.) number of compounds with known activity/inactivity, 4.) Number of compounds with unknown activity, 5.) percentage of known compounds being active and 6.) percentage of compounds with known activity readouts.


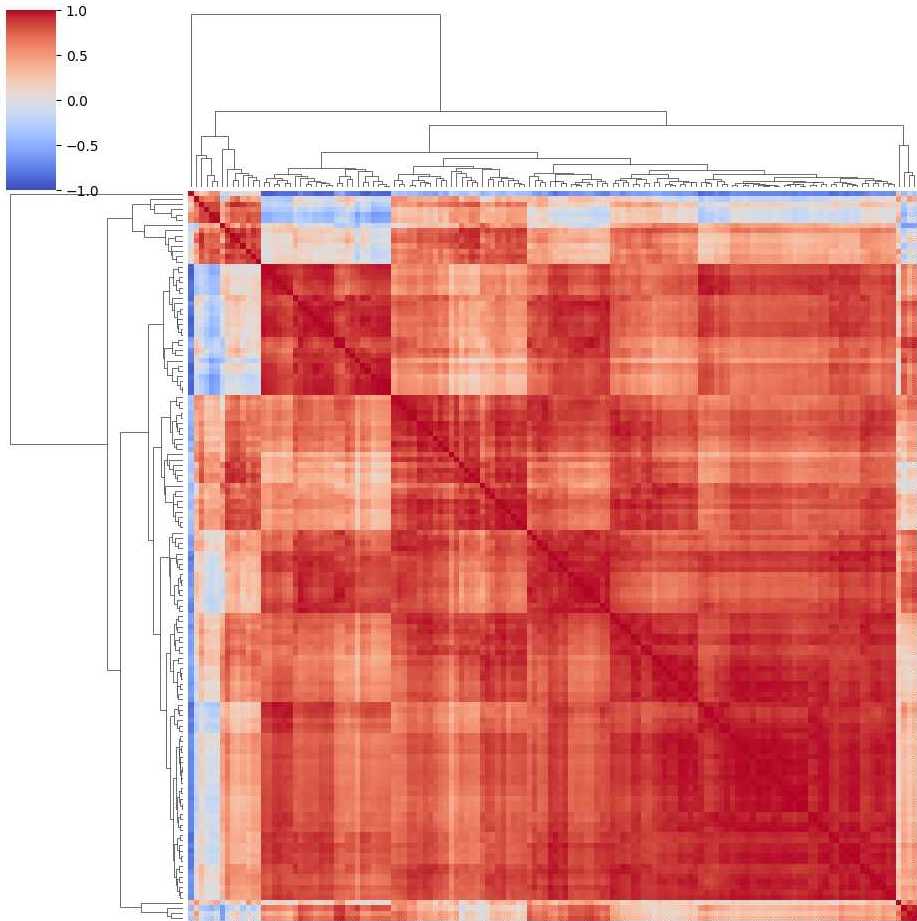

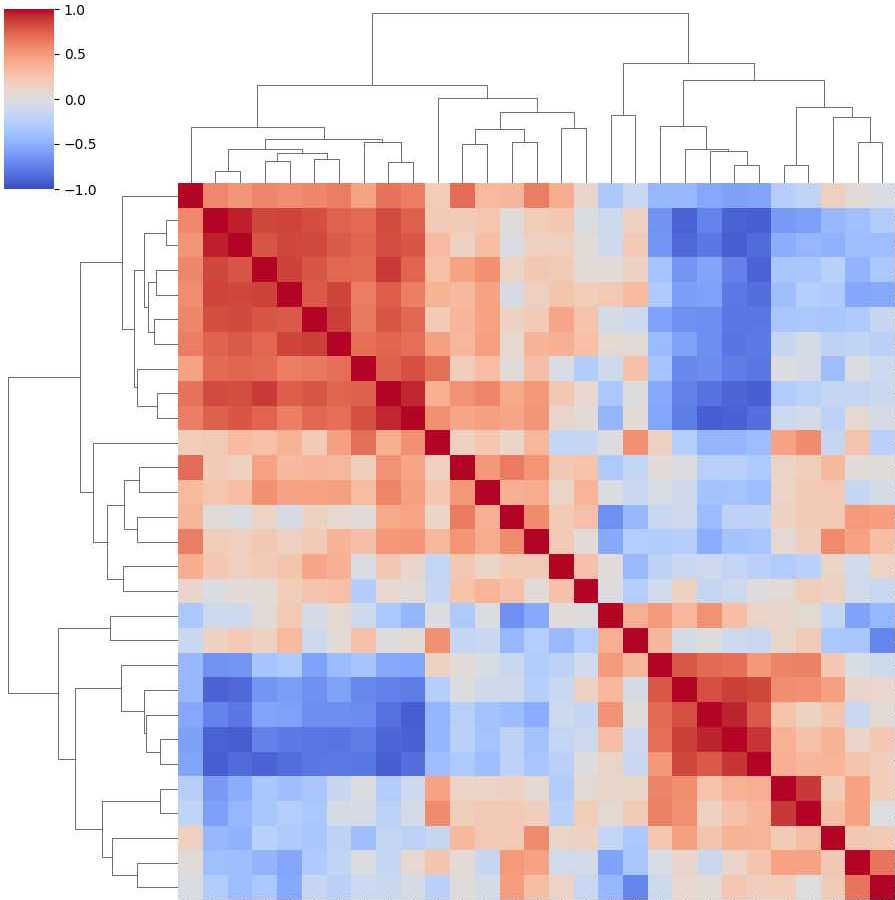


**Supplementary Figure 3 – Assay Correlation.** Cluster map plots of the inter-assay correlation between the predictions for each assay. Measured using Person correlation between the output prediction of the Whole Image Fluorescence model for the 140 internal assays (Left) and the 29 assays in the JUMP-CP & ChEMBL dataset (Right).

Dark red indicating highly correlated, light colors indicating low or no correlation and dark blue anti-correlated. (Left) The assay correlation in the internal HTS dataset, showing several groups of correlated assays in dark red, indicating that some assays are predicted to be active based on similar looking phenotypes. While many show low correlation indicating low similarity between predictions. A few show clear negative correlation, dark blue, indicating opposite prediction based on the same phenotype. The cluster maps grouping of the assay does not show any clear grouping based on assay or target type (data not shown). (Right) JUMP-CP & ChEMBL dataset shows a similar pattern to the internal data, showing several highly correlated sub-clusters, large number of assays with low correlation as well as a few anti-correlated ones.

**Supplementary Figure 4 – Benchmarking on dataset employed by Hofmarcher et al.**[**^12^**](#_ENREF_12) The boxplot illustrates the ROC-AUC performances for each assay-concentration combination in every cross-validation test-split (n=3 splits), center value defining median with boxes indicating the IQR and whiskers extending to extreme points or maximally ±1.5 × IQR., arranged by the median ROC-AUC performance. Out of 209 assays, 116 achieved ROC-AUC values of ≥0.7, 84 achieved ≥0.8, and 68 attained ≥0.9. This resulted in some extreme predictive performances of 1.0 and 0.0 for certain examples, given the utilization of only three cross-validation splits. Interestingly, while the other two datasets exhibited a steadily increasing performance trend, this dataset displayed a distinctive pattern with two sets of tasks. One set showcased very high performance, all reaching ROC-AUC values above 0.9, while the remaining sharply declined afterward, forming an elbow-like pattern. The reason behind this trend remains unclear. Source data are provided as a Source Data file.


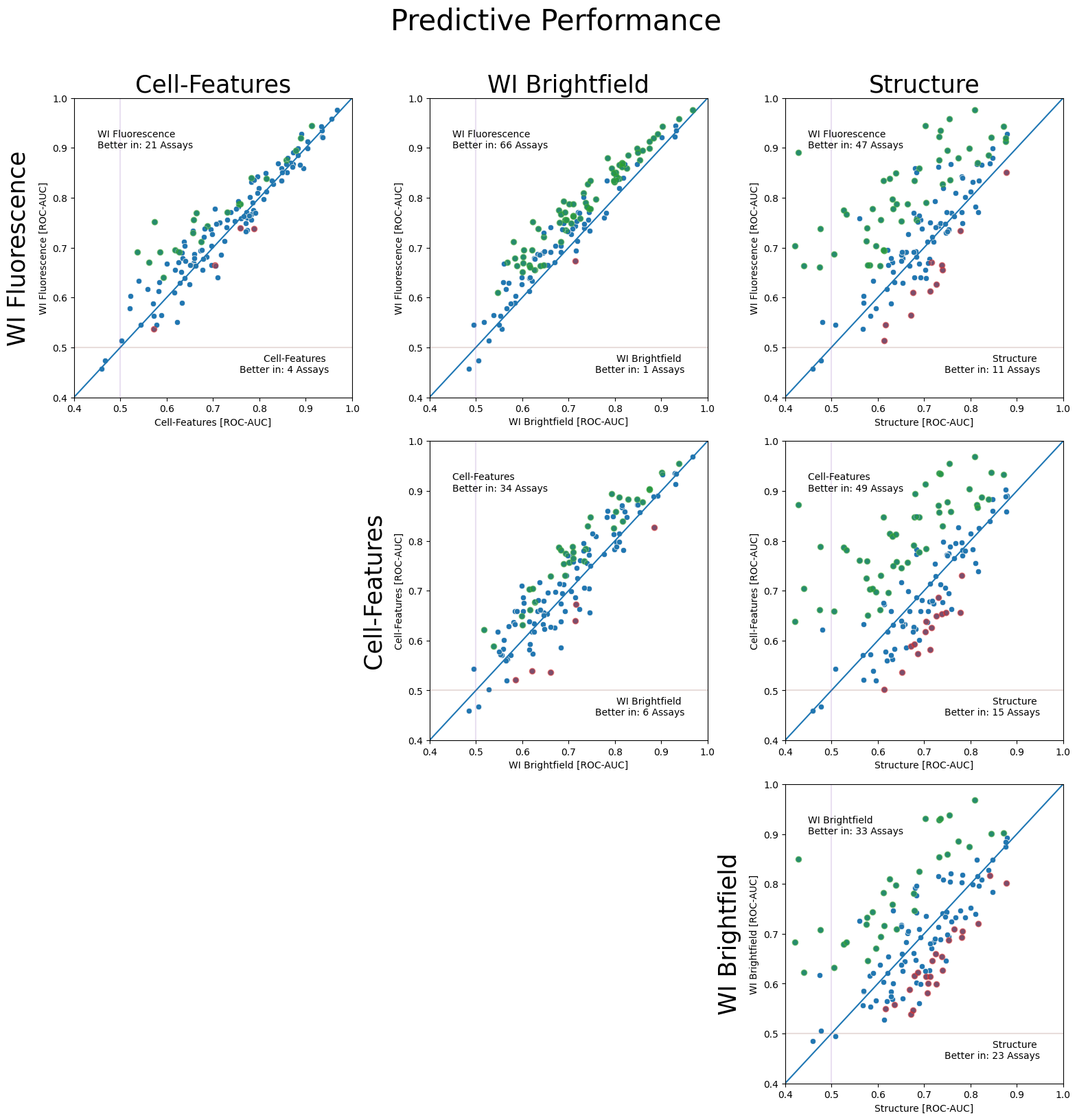


**Supplementary Figure 5 – Assay wise performance comparison between Modality types.** Comparative performance plot, representing mean assay vise performance for one modality each per axis (n=140 assays). Each dot represents an assay, with color representing if there is a significant difference in performance over 6-splits. Green – Modality on y-axis performing better, Red – Modality on x-axis and blue not showing significance. Statistical test using paired Wilcoxon signed-ranked test, with p < 0.05 set as cutoff for use of red/green coloring.


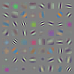

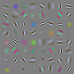


**Supplementary Figure 6 – Filter.** Grid plot of each of the convolutional filters at the input layer of the whole image fluorescent predictor model after training. Given the five input channels to the network the filters have been split into two separate images, (left) containing channels 1-3 and (right) channels 3-5.


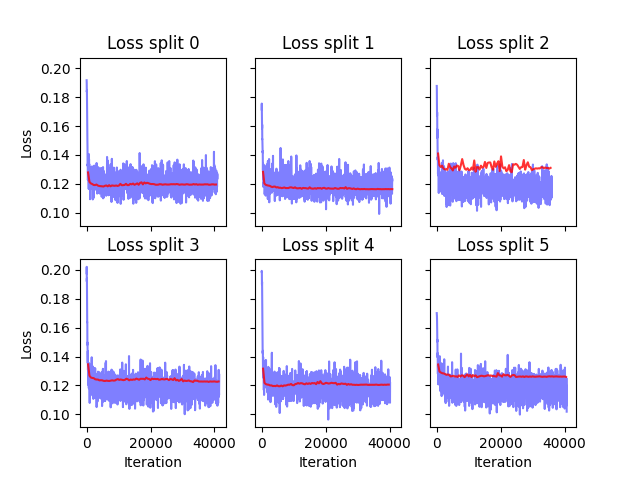


**Supplementary Figure 7 – Loss curves.** Loss curves for all six splits of the whole image fluorescent bioactivity predictor model on the publicly available JUMP-CP images. Blue curves show the training loss and red curves the validation loss.

| **Assay target** | **HTS**  **hit rate** | **ROC-AUC** | **Total number of compounds screened in follow-up** | **Number of randomly selected compounds screened in follow-up** | **Enrichment** |
| --- | --- | --- | --- | --- | --- |
| Methyltransferase | 0.88% | 0.70 ± 0.058 | 1414 | 571 | 1.6-fold |
| Polymerase | 1.14% | 0.68 ± 0.032 | 1467 | 560 | 4.6-fold |
| Oxidoreductase | 2.33% | 0.71 ± 0.093 | 1441 | 619 | 6.4-fold |
| Serine kinase | 3.78% | 0.91 ± 0.031 | 1105 | 566 | 14-fold |

**Supplementary Table 1 – Overview of in vitro validation assays.**
