# Supplementary material for: Cell Painting-based bioactivity prediction boosts high-throughput screening hit-rates and compound diversity
Source: Nat Commun. 2024 Apr 24;15:3470. doi: 10.1038/s41467-024-47171-1 (PMC11043326; doi:10.1038/s41467-024-47171-1)
Supplement: Supplementary file 2 — Reporting Summary [file 41467_2024_47171_MOESM2_ESM.pdf]

nature portfolio

Corresponding author(s): Erik Müllers

Last updated by author(s): Mar 18, 2024

Reporting Summary

Nature Portfolio wishes to improve the reproducibility of the work that we publish. This form provides structure for consistency and transparency in reporting. For further information on Nature Portfolio policies, see our Editorial Policies and the Editorial Policy Checklist.

Statistics

For all statistical analyses, confirm that the following items are present in the figure legend, table legend, main text, or Methods section.

n/a	Confirmed	
☐	☒	The exact sample size (n) for each experimental group/condition, given as a discrete number and unit of measurement
☒	☐	A statement on whether measurements were taken from distinct samples or whether the same sample was measured repeatedly
☐	☒	The statistical test(s) used AND whether they are one- or two-sided<br>*Only common tests should be described solely by name; describe more complex techniques in the Methods section.*
☐	☒	A description of all covariates tested
☐	☒	A description of any assumptions or corrections, such as tests of normality and adjustment for multiple comparisons
☐	☒	A full description of the statistical parameters including central tendency (e.g. means) or other basic estimates (e.g. regression coefficient) AND variation (e.g. standard deviation) or associated estimates of uncertainty (e.g. confidence intervals)
☐	☒	For null hypothesis testing, the test statistic (e.g. F, t, r) with confidence intervals, effect sizes, degrees of freedom and P value noted<br>*Give P values as exact values whenever suitable.*
☐	☒	For Bayesian analysis, information on the choice of priors and Markov chain Monte Carlo settings
☒	☐	For hierarchical and complex designs, identification of the appropriate level for tests and full reporting of outcomes
☒	☐	Estimates of effect sizes (e.g. Cohen's d, Pearson's r), indicating how they were calculated

Our web collection on statistics for biologists contains articles on many of the points above.

Software and code

Policy information about availability of computer code

Data collection	The code is available on github at https://github.com/cfredinh/bioactive<br>Version numbers: python==3.8 ;pytorch==1.8.1;torchvision=0.9.1;cudatoolkit=10.2.89;scikit-learn==0.24.2;matplotlib==3.4.2 ;ipykernel==5.5.5;pandas==1.2.4;ipywidgets==7.6.3;umap-learn==0.5.1;scikit-image==0.18.1;scikit-posthocs==0.8.1;albumentations==1.4.1;awscli==1.19.53;tabulate==0.8.9;colorcet==2.0.6;datashader==0.13.0;rdkit-pypi==2022.9.5;bokeh==2.3.2;holoviews==1.14.4;h5py==3.2.1;easydict==1.9;wandb==0.10.31;tqdm==4.61.0;openpyxl==3.0.7;shapely==1.7.1 ;seaborn==0.12.0;DeepProfiler
Data analysis	The code is available on github at https://github.com/cfredinh/bioactive<br>Version numbers: python==3.8 ;pytorch==1.8.1;torchvision=0.9.1;cudatoolkit=10.2.89;scikit-learn==0.24.2;matplotlib==3.4.2 ;ipykernel==5.5.5;pandas==1.2.4;ipywidgets==7.6.3;umap-learn==0.5.1;scikit-image==0.18.1;scikit-posthocs==0.8.1;albumentations==1.4.1;awscli==1.19.53;tabulate==0.8.9;colorcet==2.0.6;datashader==0.13.0;rdkit-pypi==2022.9.5;bokeh==2.3.2;holoviews==1.14.4;h5py==3.2.1;easydict==1.9;wandb==0.10.31;tqdm==4.61.0;openpyxl==3.0.7;shapely==1.7.1 ;seaborn==0.12.0;DeepProfiler

For manuscripts utilizing custom algorithms or software that are central to the research but not yet described in published literature, software must be made available to editors and reviewers. We strongly encourage code deposition in a community repository (e.g. GitHub). See the Nature Portfolio guidelines for submitting code & software for further information.

Data

Policy information about availability of data

All manuscripts must include a data availability statement. This statement should provide the following information, where applicable:

- Accession codes, unique identifiers, or web links for publicly available datasets
- A description of any restrictions on data availability
- For clinical datasets or third party data, please ensure that the statement adheres to our policy

The raw HTS datasets generated and analysed in this study are protected and are not available due to them being AstraZeneca proprietary information. The publicly available Cell Painting data used in this study are available from the JUMP consortium dataset13, CPG0016 available from the Cell Painting Gallery on the Registry of Open Data on AWS. (https://registry.opendata.aws/cellpainting-gallery/). The compound activity data used in this study are available from the ChEMBL14 database version 33 (https://www.ebi.ac.uk/chembl/). We provide a script along with a comprehensive step-by-step guide for the automated download and pre-processing of the Cell Painting/ChEMBL dataset, available at https://github.com/cfredinh/bioactive

Source data are provided as a Source Data file.

Research involving human participants, their data, or biological material

Policy information about studies with human participants or human data. See also policy information about sex, gender (identity/presentation), and sexual orientation and race, ethnicity and racism.

Reporting on sex and gender	not applicable
Reporting on race, ethnicity, or other socially relevant groupings	not applicable
Population characteristics	not applicable
Recruitment	not applicable
Ethics oversight	not applicable

Note that full information on the approval of the study protocol must also be provided in the manuscript.

Field-specific reporting

Please select the one below that is the best fit for your research. If you are not sure, read the appropriate sections before making your selection.

[x] Life sciences   [ ] Behavioural & social sciences   [ ] Ecological, evolutionary & environmental sciences

For a reference copy of the document with all sections, see nature.com/documents/nr-reporting-summary-flat.pdf

Life sciences study design

All studies must disclose on these points even when the disclosure is negative.

Sample size	The sample size used was not determined based on any statistical method, all data available was included.
Data exclusions	The creation of dataset is described in the materials and methods section. No data were excluded from the analysis.
Replication	Data splits, randomization, and replication are detailed in results and materials and method section. All replications are described in the manuscript text and figure legends and gave coherent results.
Randomization	Data splits, randomization, and replication are detailed the results and materials and method section. For the wet lab experiments ("follow-up screening") randomized plate layouts were employed.
Blinding	The individuals performing the wet lab experiments ("follow-up screening") were not aware of the results of the bioactivity prediction i.e., only the compound numbers were shared for screening.

Reporting for specific materials, systems and methods

We require information from authors about some types of materials, experimental systems and methods used in many studies. Here, indicate whether each material, system or method listed is relevant to your study. If you are not sure if a list item applies to your research, read the appropriate section before selecting a response.

Materials & experimental systems

n/a	Involved in the study
☒	☐ Antibodies
☐	☒ Eukaryotic cell lines
☒	☐ Palaeontology and archaeology
☒	☐ Animals and other organisms
☒	☐ Clinical data
☒	☐ Dual use research of concern
☒	☐ Plants

Methods

n/a	Involved in the study
☒	☐ ChIP-seq
☒	☐ Flow cytometry
☒	☐ MRI-based neuroimaging

Eukaryotic cell lines

Policy information about cell lines and Sex and Gender in Research

Cell line source(s)	U-2 OS acquired from ATCC https://www.atcc.org/products/htb-96
Authentication	All cell lines were directly acquired from ATCC.
Mycoplasma contamination	All cell lines were tested negative for mycoplasm contamination.
Commonly misidentified lines (See ICLAC register)	None of the cell lines used are known to be commonly misidentified lines

Plants

Seed stocks	not applicable
Novel plant genotypes	not applicable
Authentication	not applicable
